# Methylcellulose-Directed Synthesis of Nanocrystalline Zeolite NaA with High CO_2_ Uptake

**DOI:** 10.3390/ma7085507

**Published:** 2014-07-28

**Authors:** Dilshod Shakarova, Arto Ojuva, Lennart Bergström, Farid Akhtar

**Affiliations:** 1 Department of Materials and Environmental Chemistry, Stockholm University, Stockholm 10691, Sweden; E-Mails: dilshoda.shakarova@mail.ru (D.S.); arto.ojuva@mmk.su.se (A.O.); Lennart.bergstrom@mmk.su.se (L.B.); 2 Berzelii Center EXSELENT on Porous Materials, Stockholm University, Stockholm 10691, Sweden; 3 Division of Materials Science, Luleå University of Technology, Luleå 971 87, Sweden

**Keywords:** zeolite NaA, nanocrystals, hydrothermal synthesis, carbon dioxide adsorption

## Abstract

Zeolite NaA nanocrystals with a narrow particle size distribution were prepared by template-free hydrothermal synthesis in thermo-reversible methylcellulose gels. The effects of the amount of methylcellulose, crystallization time and hydrothermal treatment temperature on the crystallinity and particle size distribution of the zeolite NaA nanocrystals were investigated. We found that the thermogelation of methylcellulose in the alkaline Na_2_O-SiO_2_-Al_2_O_3_-H_2_O system played an important role in controlling the particle size. The synthesized zeolite nanocrystals are highly crystalline, as demonstrated by X-ray diffraction (XRD), and scanning electron microscopy (SEM) shows that the nanocrystals can also display a well-defined facetted morphology. Gas adsorption studies on the synthesized nanocrystalline zeolite NaA showed that nanocrystals with a size of 100 nm displayed a high CO_2_ uptake capacity (4.9 mmol/g at 293 K at 100 kPa) and a relatively rapid uptake rate compared to commercially available, micron-sized particles. Low-cost nanosized zeolite adsorbents with a high and rapid uptake are important for large scale gas separation processes, e.g., carbon capture from flue gas.

## 1. Introduction

Zeolites are porous aluminosilicates with a high surface area and a high thermal stability and are used in applications such as catalysis [1,2], ion exchange [3,4] and in separation and purification processes [5,6]. Currently, the potential use of low-cost zeolites for carbon capture is to mitigate the anthropogenic release of carbon dioxide, e.g., combustion of fossil fuels is attracting a significant interest [7,8,9]. The CO_2_ adsorption in various well-known zeolites has been extensively studied [10], and among them, the zeolites of framework types FAU (Faujasite) (X, Y) and LTA (Linde Type A) (A) display interesting combination of high CO_2_ adsorption capacity and high selectivity to, e.g., N_2_. Zeolite A is a suitable adsorbent for small molecules, due to the apertures of its eight-membered ring windows that are comparable in size with the kinetic diameters of N_2_ and CO_2_ [11,12].

Low-cost nanosized zeolite adsorbents that can combine a high adsorption capacity and good selectivity with rapid adsorption kinetics are of importance for carbon capture from flue gas. While the capacity and selectivity are primarily determined by the chemical and topological features of the pores, the uptake kinetics can also be controlled by reducing the diffusion length, e.g., by decreasing the particle size. A number of approaches have been developed to synthesize zeolite nanocrystals with and without the use of structure-directing agents [13,14,15,16,17]: Valtchev *et al.* showed that zeolite A nanocrystals with a size of 100–300 nm could be obtained by a slow (three days) room temperature, template-free synthesis in the system of Na_2_O-SiO_2_-Al_2_O_3_-H_2_O [14]. However, the well-faceted crystal morphology was obtained for crystals averaging 400–500 nm after prolongation of synthesis time to 10 days. Zhang *et al.* extended the work on room temperature, template-free synthesis and reported that the crystallization time, ageing time of precursor solution, stirring time and type of silica/alumina source influenced the morphology and particle size of zeolite A crystals [15]. However, the zeolite A crystals showed a broad particle size distribution, and only a mean crystal size of 0.4 μm could be obtained by tuning the synthesis parameters. Confined space synthesis of zeolite nanocrystals was induced by Madsen and Jacobsen [18] and has been expanded using both hard (mesoporous carbon) [19] and soft gels, e.g., chemically cross-linked polyacrylamide [20] and physical hydrogels of methyl cellulose [21] and chitosan [22]. Confined space synthesis of zeolites in the synthesis system of Na_2_O-SiO_2_-Al_2_O_3_-H_2_O in thermoreversible polymer hydrogels has shown a controlled zeolite growth rate and produced size-controllable zeolite crystals with a narrow particle size distribution. Moreover, thermoreversible polymers can be removed by washing, which makes the recovery simple, and the obtained crystals can be dispersed in a solvent [21].

In the present work, we have successfully prepared nanocrystalline zeolite A and systematically investigated how the amount of methylcellulose, temperature and heating time control the particle size distribution, crystallinity and morphology of the nanocrystals. The crystal growth was related to the hydrogel formation process in the Na_2_O-SiO_2_-Al_2_O_3_-H_2_O/methylcellulose system, which was followed by rheology. The CO_2_ adsorption capacity and the uptake kinetics have been evaluated for the synthesized nanocrystalline zeolite NaA and compared with conventional micron-sized crystals, and the potential for using nanosized zeolites as an adsorbent for the capture of CO_2_ has been discussed.

## 2. Results and Discussion

**Figure 1:**
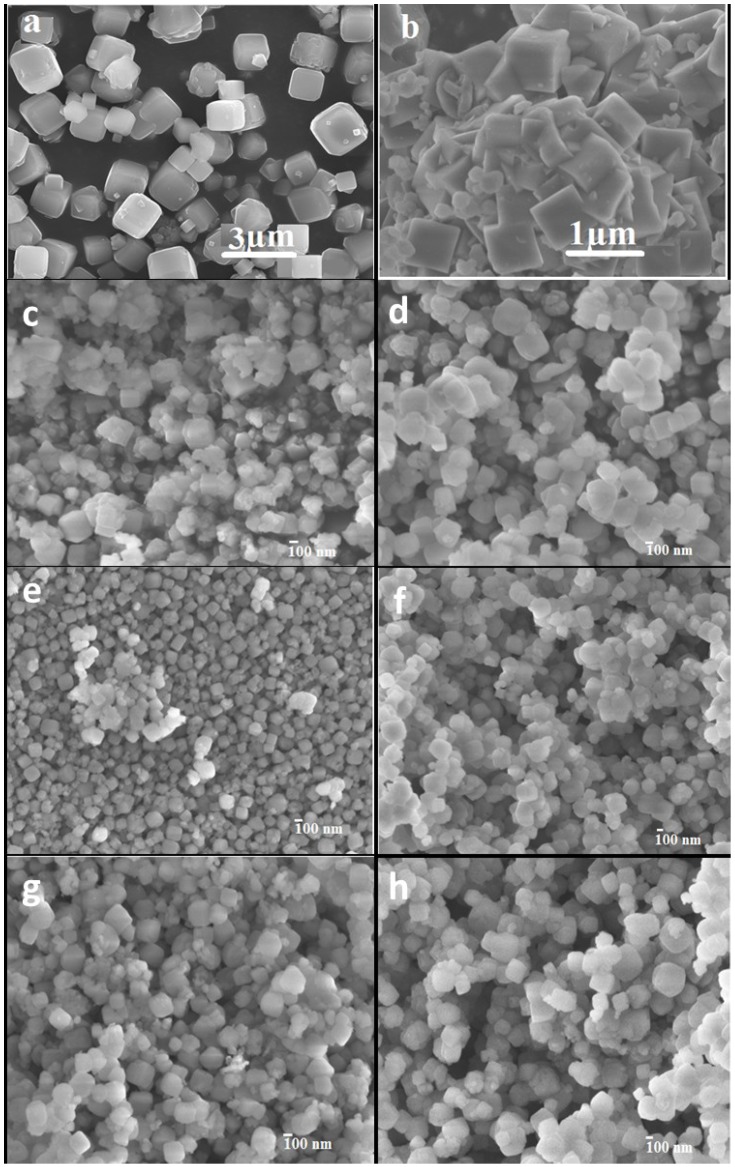
Scanning electron microscope(SEM) images of the nanosized zeolites synthesized with different amount of methylcellulose (MC): (**a**) conventional zeolite 4A; (**b**) Z0; (**c**) NZ010; (**d**) NZ020; (**e**) NZ040; (**f**) NZ060; (**g**) NZ080; (**h**) NZ100.

Nanocrystalline zeolite A has been hydrothermally synthesized in solutions containing thermo-reversible methylcellulose. Table 1 summarizes how the particle size, determined by dynamic light scattering (DLS), varies with the amount of methylcellulose (MC) added. For comparison, materials synthesized without any MC added (Z0) and a commercially available zeolite A powder (NaA) were also included. The DLS measurements indicate a slightly smaller change in the average particle size than the scanning electron micrographs indicate, e.g., NZ040 and NZ080 are reported by DLS as having an average size of 100 ± 20 nm and 150 ± 50 nm, respectively, but Figure 1e (NZ040) and 1g (NZ080) show a larger size variation. This might be due to presence of a mix of small and large crystals, which explains the appearance of seemingly large particles in the micrographs. Due to broad particle size distribution and different sampling procedure, the differences in the particle size determination between the two techniques will be inevitable [23].

**Table 1:** Experimental conditions for the synthesis of nanosized zeolite A particles and the corresponding properties.

Sample	Mass of MC(g)/% of PSS ^a^	Mean particle diameter (nm) ^b^	ξ-potential (mV) ^c^	S_ext/_(in m^2^ g^−1^) ^d^
Z0	0	1500 ± 500	−39	12
NZ010	0.1/0.9	250 ± 100	−40	36
NZ020	0.2/1.8	220 ± 60	−43	37
NZ040	0.4/3.5	100 ± 20	−48	61
NZ060	0.6/5.2	150 ± 30	−47	57
NZ080	0.8/6.8	150 ± 50	−45	55
NZ100	1.0/8.3	200 ± 20	−44	50
4A	-	3000 ± 500	−39	2

^a^ PSS = precursor solution system; ^b^ DSL = dynamic light scattering; ^c^ The zeta potential of dispersed zeolite Micron- and nanocrystals were determined with a Zetasizer Nano NS at 25 °C; ^d^ The external surface area of micron- and nano-sized zeolites was determined by nitrogen adsorption at 77 K.

The MC gel was easily removed after zeolite crystallization by simple washing at room temperature. We found that the zeta potential changed slightly from −47 ± 12 mV for synthesized nanosized particles to −40 ± 1.9 mV for micron-sized particles of zeolite A. The scanning electron microscopy images of the synthesized particles corroborate the DLS measurements, showing that the addition of no MC (Z0) results in crystals with a size exceeding one micrometer, and the smallest zeolite A crystals with a size of around 100 nm are prepared with an addition of 0.4 g MC (NZ040). Figure 1 shows that the nanozeolite crystals displayed a well-defined cubic morphology.

The NaA zeolite nanocrystals were investigated by N_2_ uptake measurements at 77 K. The zeolite nanocrystals were degassed at 573 K for 10 h to ensure complete removal of volatile components prior to the N_2_ adsorption measurement. The N_2_ adsorption and desorption isotherm of nanosized zeolite NZ040 (Figure S1; see the Supplementary Information) are of Type II, indicating a non-porous powder, which suggests that the pore openings are too small for N_2_ to enter at 77 K [14,24,25,26]. Thus, the measured BET (Brunauer-Emmet-Teller) surface area in Table 1 represents the external surface area of the zeolite A nanocrystals. Rezaei *et al.* [27] have reported that external surface area per unit volume is one of the important parameters and determines the mass transfer in an adsorbent. In our case, the nanocrystals displayed a high external surface area of 61 m^2^*/*g, which is significantly higher compared to the external surface area of 2 m^2^/g of micron-sized zeolite A crystals.

**Figure 2:**
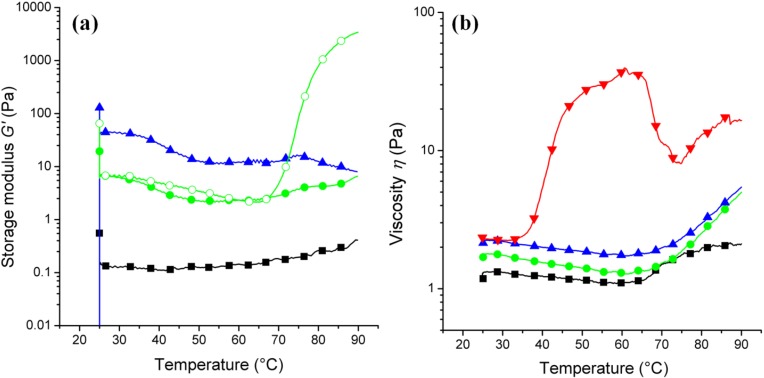
(**a**) Influence of the methylcellulose concentration and the temperature on the relative viscosity for the precursor solution mixed with 0% (Z0, black square), 3.5% (NZ040, green filled circles), 6.8% (NZ080, blue triangle up) and 3.5% (pure MC at pH = 11, green open circles) of methylcellulose, determined from oscillatory measurements at 1 Hz. (**b**) Gel formation determined from rotational measurements at 1 Hz: PSS (black squares), 3.5% (NZ04, green circles), 6.8% (NZ080, blue triangles up) and 3.5% (pure MC at pH = 11, upside-down red triangles) (samples with g/100 mL water or precursor solution).

We have studied the influence of temperature on the rheological properties of MC/precursor solution system (PSS) solutions with different concentrations (3.5%–6.8%) of MC in PSS (Figure 2a). Methylcellulose (MC) completely dissolves in water and forms a homogeneous hydrogel upon heating. The gelling of MC molecules is driven by dehydration, where the MC chains form a network. Pure MC gels form between 50–70 °C. It should be mentioned that we have used a high molecular weight (MW) MC, MW = 87,000, for the synthesis of nanozeolites from the precursor solution, which is more than six times higher than the MC used previously by Wang *et al.* [21]; MW = 14,000. Polymers with a higher MW are expected to form gels at lower concentrations (from 1.8%–5.2%).

The gelation in the alkaline precursor solution (Figure 2b) is much less pronounced than in the pure MC solutions. This can be related to an increase in the onset temperature of gelation, which may coincide with the temperature range for the break-down of the hydrogel. We have successfully controlled the size of the zeolite nanocrystals using different amounts of MC. The zeolite nanocrystals that were grown in the more concentrated (3.5%–5.2%) MC hydrogel networks remain very small (<200 nm), whereas crystals precipitated with little or no MC could grow much larger (Figure 1).

**Figure 3:**
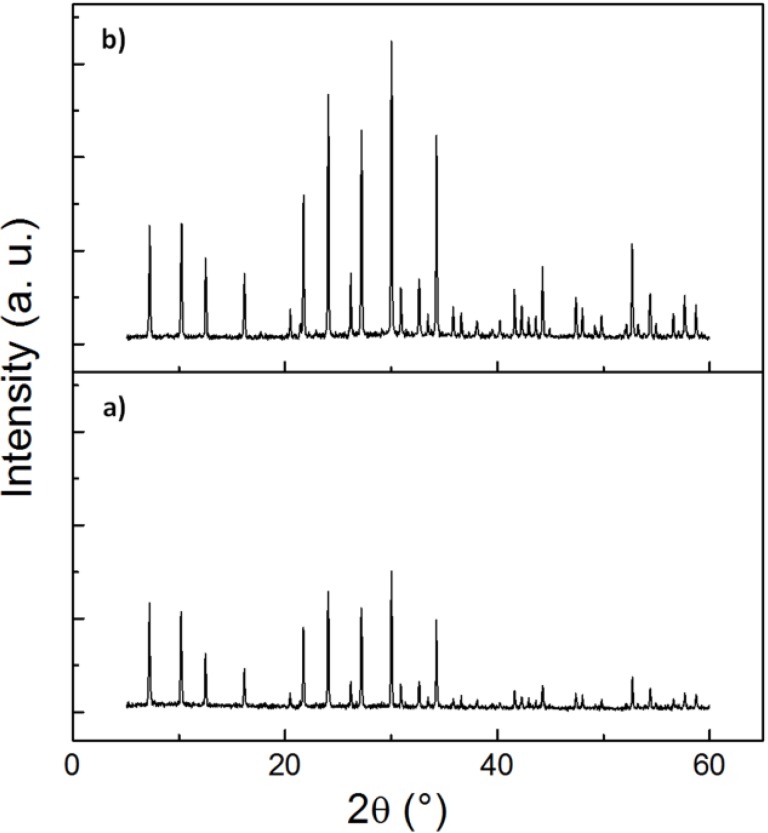
X-ray diffraction patterns of the nanosized zeolites, methylcellulose removed from zeolite matrix by simply washing and calcination: (**a**) NZ040 after washing and (**b**) NZ040 after calcination at 773 K for 6 h.

**Figure 4:**
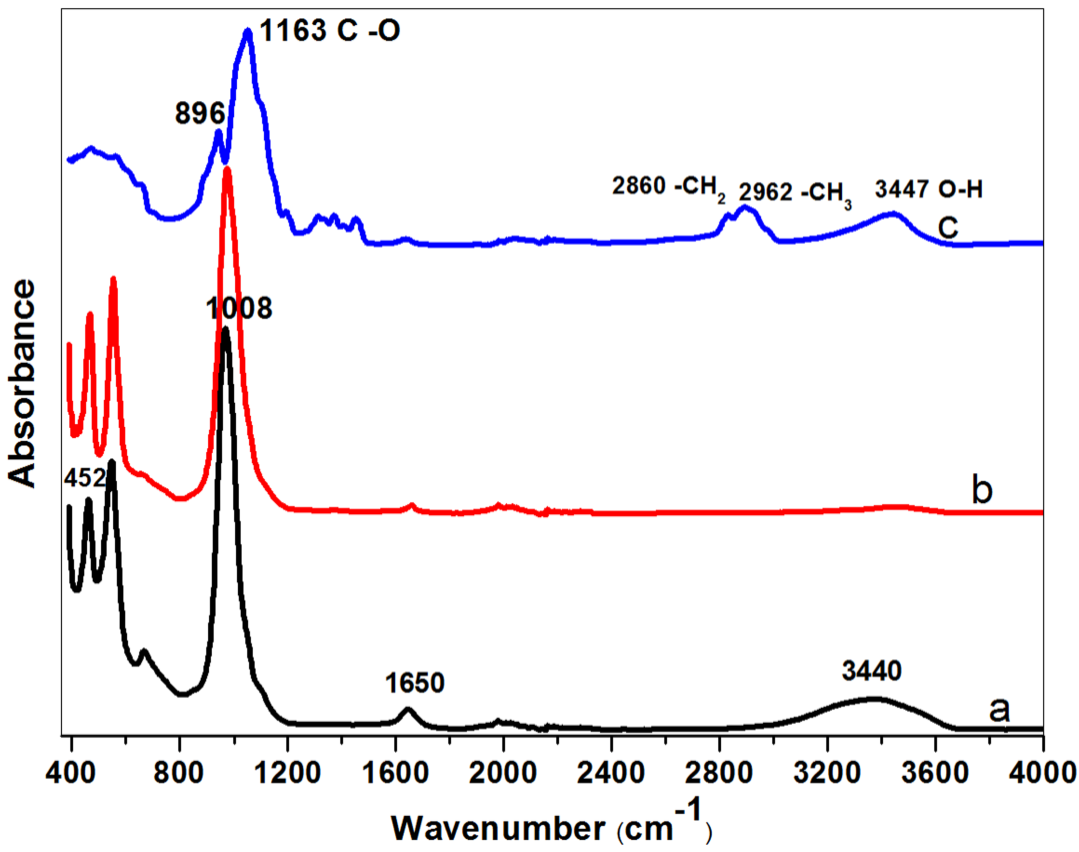
FTIR (Fourier transform infrared) spectra of nanometer-sized NaA samples and MC: methylcellulose removed by (a) simply washing (NZ040); (b) calcination (NZ040); and (c) pure MC.

The crystallinity and phase mixtures have been determined by the Rietveld method from the powder X-ray diffraction (XRD) pattern, using least-squares fitting. The XRD diffractograms in Figure 3 and Figure S2 (see the Supplementary Information) show that the nanosized zeolite NaA are highly crystalline and phase pure. Although the absolute peak intensities vary due to the preparation of samples for XRD (e.g., the amount of powder on the silicon plate varies from sample to sample), we find that calcination does not result in any significant change in the peak position or the width of the peaks, confirming the high crystallinity of the as-synthesized powders. Figure S3 (see the Supplementary Information) also shows that the crystallinity of the NZ040 powders is similar to the crystals synthesized without any MC added and a commercial zeolite A powder. Rietveld refinement of the powder X-ray diffraction data confirmed that the crystallinity of all synthesized nanozeolite 4A powders was greater than 90%, and the crystals with a size <220 nm were >95% crystalline. Determination of the elemental composition using energy-dispersive X-ray spectroscopy (EDXS) corroborated that all obtained samples are indeed chemically pure nanosized zeolite NaA (Figure S4). Figure 4 shows the Fourier-transform infrared (FTIR) spectra of nanocrystalline NaA zeolites after being heated at 573 K and pure MC powder at 373 K overnight. The IR bands appear at nearly identical wavenumbers for these powders. We find that pure MC has distinctive absorption bands related to O–H stretching at 3447 cm^−1^, –CH_3_ stretching of the anhydroglucose unit at 2962 cm^−1^, –CH_2_– stretching of the anhydroglucose unit at 2860 cm^−1^, C–O carbonyl stretching in the anhydroglucose unit at 1643 cm^−1^, C–OH in-plane bending at 1440 cm^−1^, CH_3_ symmetric bending at 1375 cm^−1^, C–O stretching from asymmetric oxygen bridge at 1163 cm^−1^ and ring stretching at 896 cm^−1^. These values are consistent with the previous report by Rimdusit *et al* [28]. The broad band at about 3440 cm^−1^ and the band around 1650 cm^−1^ in the as-synthesized, un-calcined nanozeolite can be attributed to water bound in the zeolitic structure. The bands at about 452 cm^−1^ are due to internal vibrations of (Si, Al)O_4_ tetrahedra of NaA, whereas the band at about 1008 cm^−1^ is due to asymmetrical vibrations related to (Si, Al)O_4_ tetrahedra of NaA [15,29]. Overall, the FTIR spectra of nanocrystalline zeolite A uncalcined sample is matched well with the typical FTIR absorption peaks of calcined nanosized zeolites, but the calcined samples have lost most of their O–H groups. It is well known that the hydroxyl groups are important for the chemistry of zeolite materials [30]; therefore, to remove MC from zeolite by a mild sample washing is preferred compared to calcination. Additionally, the MC has indeed not been incorporated in the zeolite crystals, as was shown in the FTIR spectra.

**Figure 5:**
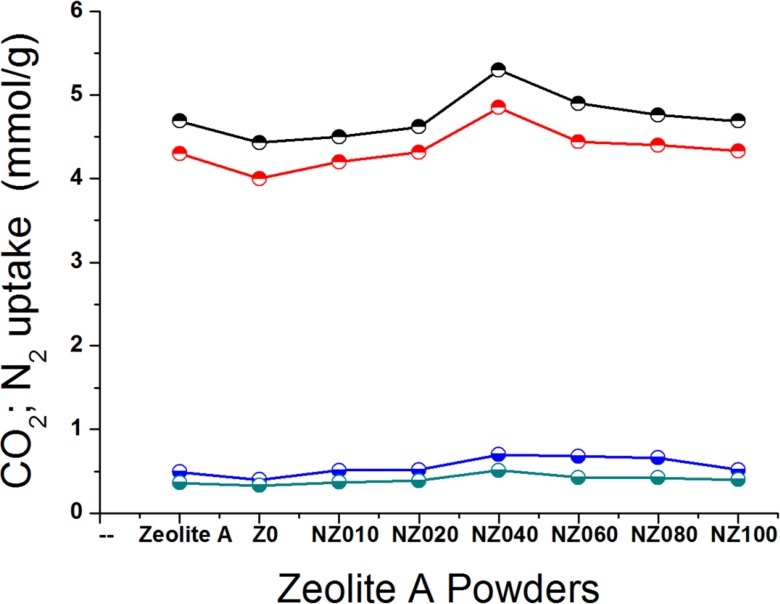
CO_2_ and N_2_ uptake of different nano- and micron-sized zeolites at 273 and 293 K; 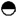, CO_2_ uptake at 273 K; 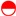, CO_2_ uptake at 293 K; 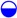, N_2_ uptake at 273 K; 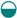, N_2_ uptake at 293 K.

The CO_2_ adsorption-desorption isotherms at 273 and 293 K for five different nanosized zeolites and two micron-sized zeolites are shown in Figure 5. The amount of CO_2_ adsorbed in the micron-sized powders Z0 and Zeolite A were lower than that for the smaller particles, especially the highly crystalline NZ040, NZ060 and NZ080. The uptake of N_2_ was also determined at 273 and 293 K for all nano- and micron-sized zeolites (Figure 5). The low uptake of N_2_ in comparison with CO_2_ makes zeolite 4A a good candidate for the separation of those gases.

Table 2 shows the diffusion coefficients derived from the time-dependent uptake of CO_2_ on the micron- and nano-sized zeolite A. The diffusion time constants (*D*_c_/*r*_c_^2^, s^−1^, where *D*_c_ is the intercrystalline diffusivity and *r*_c_ is the radius of the particle) were calculated from the slope of a linear plot of ln[1 − (*m_t_*/*m*_∞_)] (where *m_t_* is the uptake at time *t* and *m*_∞_ is the maximum uptake) *versus* *t* (time) at a given pressure for uptakes greater than 70%. The linear relation between the uptake ln[1 − (*m_t_*/*m*_∞_)] and time (t) may also be applicable at initially small amounts of CO_2_ uptake (see Supplementary Figure S5), which indicates that the adsorption kinetics are controlled by a surface resistance to diffusion [31,32]. The diffusion time constant (*D*_c_/*r*_c_^2^, Table 2) does not improve significantly with decreasing NaA particle size, depicting that the apparent intercrystalline diffusivity (*D*_c_) would decrease with a decrease in particle size. Therefore, a surface resistance for carbon dioxide diffusion in Zeolite A nanocrystals may be present due to an amorphous layer around the crystals or in a distorted crystal structure layer around the crystals [33]. The uptake kinetics (Table 2, see Supplementary Figure S6) show that the synthesized NaA zeolite displays relatively faster CO_2_ adsorption compared to micron-sized zeolite A. However, we did not find the expected dependence of the diffusion time constant on the square of the radius of the crystals. Our synthesized nanocrystals show high crystallinity and high CO_2_ adsorption capacity during equilibrium uptake. Therefore, we can attribute this to the presence of skin layer resistance and thermal effects, where an increase in temperature influences the adsorption kinetics. The role of thermal effects have been shown in several zeolitic system [34,35]. We determined the diffusion time constants of CO_2_ uptake, where the zeolite powder was placed on aluminum foil to remove the heat of adsorption. We found significant improvement in the CO_2_ adsorption kinetics on zeolite A (Table 2). We suggest that a better process design for the removal of the heat of adsorption is important to obtain the rapid adsorption kinetics on nano zeolite 4A crystals.

**Table 2:** CO_2_ adsorption kinetics of the different nano- and micron-sized zeolite (35 mg).

Zeolite	Zeolite A	Z0	NZ010	NZ020	NZ040	NZ060	NZ080	NZ100
Diffusion time constant (*D*/*R*^2^)	−0.0039/−0.0028 *	−0.0035	−0.0036	−0.0038	−0.0033/−0.0024 *	−0.0033/−0.0024 *	−0.0039	−0.0036

* Results from zeolite powders placed on aluminum foil to remove the heat of adsorption.

## 3. Experimental Section

For the synthesis of nanosized zeolite A, we adapted the confined space synthesis described by Wang *et al.* [21] and modified it significantly to optimize and tailor the size of nano-zeolite particles. A Na_2_O-SiO_2_-Al_2_O_3_-H_2_O precursor solution system (PSS) was prepared by mixing 27.0 g of SiO_2_ (HS-30 Ludox, Sigma Aldrich Chemie GmbH, Munich, Germany), 10.9 g of NaAlO_2_ (Nalco 680, Sigma Aldrich Chemie GmbH) and 20.0 g of NaOH (99% pure, Sigma Aldrich Chemie GmbH) in 161 g of double-deionized water (18.5 μS/cm at 25 °C). The PSS (precursor solution system) was sealed in a 200-mL polypropylene bottle and aged for 20 h at 298 K under vigorous stirring. Then, 16.5 g of the aged PSS was transferred into a 50 mL polypropylene bottle and stirred at 313 K for approximately 15 min before the addition of the methylcellulose (MC) powder.

The MC powder (Methocel^®^ 90 HG, MW 87000, Sigma Aldrich Chemie GmbH) had been dried overnight in a vacuum at 333 K, cooled down in a desiccator and then added in various amounts into the PSS under vigorous manual stirring to give a thick, homogeneous white paste. The amount of added MC, 0.10, 0.20, 0.40, 0.60, 0.80 and 1.00 g, was used to denote the products as NZ010, NZ020, NZ040, NZ060, NZ080 and NZ100, respectively. The reference solution with no added MC was named Z0. After stirring, the mixtures were sealed and kept at 278 K for 5 h to ensure complete dissolution of the methylcellulose. The obtained mixtures were then kept at 355 K for an additional 3 h in order to synthesize zeolite crystals. The products (a mixture of zeolite A crystals and MC) were washed several times with deionized (DI) water, followed by centrifugation at 6000 rpm for 10 min. The precipitate was dispersed in DI water by sonication and centrifuged again. This procedure was repeated, until the pH value of the supernatant dropped below 8, after which, the precipitate was dried at 373 K overnight. The zeolite nanocrystals were collected by washing away the water-soluble methylcellulose at room temperature, followed by calcination by heating the powder at 1 K/min to 773 K and kept there for 6 h. The properties of the different products are presented in Table 1.

In order to better understand the gelation mechanism, we carried out rheological measurements at various temperatures with Paar Physica MCR301 (Anton Paar GmbH, Graz, Austria) using a concentric cylinder geometry at 1 s^−1^ for the viscosity measurements and a cone-plate at 1 Hz for the oscillatory viscoelastic measurements. The temperature was increased from 293 to 363 K at 1 K/min by a Peltier module.

Prior to use, the MC polymer was dried at 323 K for 24 h to remove any moisture. The MC solutions with a concentration of 0.9–8.3 wt% were prepared with deionized water. For the comparison, rheological properties of pure double distilled water (DDW), pure methylcellulose (MC) solutions and MC/PSS (Na_2_O-SiO_2_-Al_2_O_3_-H_2_O) were investigated at pH 11. The gelation of aqueous solutions of a methylcellulose was studied at pH 7 and 11. The pH of the DDI was adjusted by 0.1 M NaOH before the addition of MC. The methylcellulose solutions were prepared by vigorous stirring to obtain a homogeneous clear solution. The solutions were de-aired in a vacuum and transferred into the cell of the rheometer Paar Physica MCR301 to study gelation in these solutions on heating.

In order to confirm the crystallinity of the synthesized powders, we used powder X-ray diffraction (PXRD). An X’PERT-PRO PANalyical powder diffractometer with an X’Celerator detector (PANalytical, Almelo, The Netherlands) (CuK_α1_ radiation, λ = 1.5418 Å) was used at 45 kV, 40 mA settings. The powders were ground using a mortar and pestle and suspended in ethanol on a specimen holder and dried. For quantification of the crystallinity of the 4 A powders, a mixture of zeolite NaA and NaX powders (total zeolite amount 50–100 mg, at approximately 1:1 proportions) was weighed and suspended in ethanol on a zero-background specimen holder and dried. Then, the powder mixture was measured in reflection mode using a 20-mm fixed mask and automated divergence slits irradiating a constant length of 10 mm for 2.5 h between 5.0–100.0° 2θ. The data were converted into fixed slit data using the software X’Pert HighScore Plus and quantified using Rietveld refinement (program FullProf) to determine the ratio of crystalline 4 A to 13 X, as described in earlier work [36].

Thermogravimetric (TG) analysis was carried out in dry air up to 1073 K using a Perkin Elmer TGA-7 instrument (PerkinElmer, Waltham, MA, USA) at a heating rate of 10 °C min^−1^. The particle size and morphology of the nanosized zeolites were investigated using a JEOL JSM-7000F (JEOL, Tokyo, Japan) scanning electron microscope (SEM) operating at an acceleration voltage of 2–5 kV. Elemental analyses of the synthesized powders were determined using energy dispersive X-ray spectroscopy (EDXS) on the JSM-7000F microscope. EDXS data were recorded from 4–15 distinct spots or areas per sample at a 10-kV accelerating voltage; see Figure S4. The analyzed and nominal composition generally agreed within 10%, which is within the uncertainties of EDXS measurements. Fourier transform infrared (FTIR) spectra were obtained on a Varian 670 FT-IR (Agilent, Santa Clara, CA, USA) in the wave number range of 4000–400 cm^−1^.

The zeta potential (ξ) and the average particle size were measured by dynamic light-scattering (DLS) measurements at 298 K on a Zetasizer Nano NS (Malvern Instruments, Worcestershire, UK) on dilute suspension (0.1 wt%) in de-ionized water. Dilute suspensions containing 0.1 wt% of zeolite were ultrasonicated for 10 minutes. Then, an aliquot of the dilute suspension was placed into a 5-mL cuvette, and each suspension was measured four times. The pH was measured using a sevenMulti instrument equipped with an Inlab expert NTC 30 electrode (Mettler-Toledo AB, Stockholm, Sweden).

N_2_ adsorption and desorption measurements were performed on a Micromeritics ASAP2020 (Micromeritics Instrument Corporation, Norcross, GA, USA) instrument at 77 K. The specific surface area was calculated using the BET model within the 0.05–0.15 *p*/*p*_o_ relative pressure region. Prior to measurement, the samples were pre-treated at 573 K under a dynamic vacuum (1 × 10^−4^ Pa) for 10 h. CO_2_ and N_2_ adsorption-desorption isotherms at 273 and 293 K were measured using a Micromeritics ASAP2020 (Micromeritics Instrument Corporation, Norcross, GA, USA) device with same procedure as described above. The kinetics of adsorption of CO_2_ on the micron-sized and nanosized zeolite A have been investigated by a thermogravimetric analyzer (TGA) using a Setaram TAG 24 instrument (SETARAM Instrumentation, Caluire, France).

The EDXS data were recorded from 4–15 distinct spots or areas per sample at a 10-kV accelerating voltage; see Figure S4. The analyzed and nominal composition generally agreed within 10%, which is within the uncertainties of EDXS measurements.

## 4. Conclusions

We have successfully synthesized nanosized zeolite NaA with thermoreversibly polymerizing methylcellulose (MC) hydrogel. The hydrogel significantly constricted the crystal growth of the zeolites, thus preventing their growth over a few hundred nanometers, depending on the hydrogel concentration. It was found that the optimum concentration for MC of a molecular weight of 87,000 in water was between 3.5%–6.8%; a lower concentration did not form a hydrogel, which was dense enough, and a higher concentration flocculated too easily.

The MC was found to synthesize highly crystalline nanosized zeolite NaA crystals without any additional chemicals or structure-directing agents. Crystal sizes ranging from 100–350 nm were obtained from Na_2_O-SiO_2_-Al_2_O_3_-H_2_O/MC solution at 355 K in 3 h. This study was aimed to advance the cost-effective synthesis methods of highly-active nanosized zeolites. The environmentally-friendly process reduces the cost of synthesis, and the affordable MC is easily removed from the system without calcination, thus also reducing the energy demands of the synthesis. The synthesized nanocrystals of zeolite NaA exhibited high capacity for carbon dioxide adsorption.

## Supplementary Files

Supplementary File 1
