# Supplementary material for: Methylcellulose-Directed Synthesis of Nanocrystalline Zeolite NaA with High CO2 Uptake
Source: Materials (Basel). 2014 Jul 28;7(8):5507–19. doi: 10.3390/ma7085507 (PMC5456199; doi:10.3390/ma7085507)

Supplementary Information

Figure S1. The nitrogen adsorption and desorption isotherms of nanosized zeolite (NZ040) measured at 77 K.

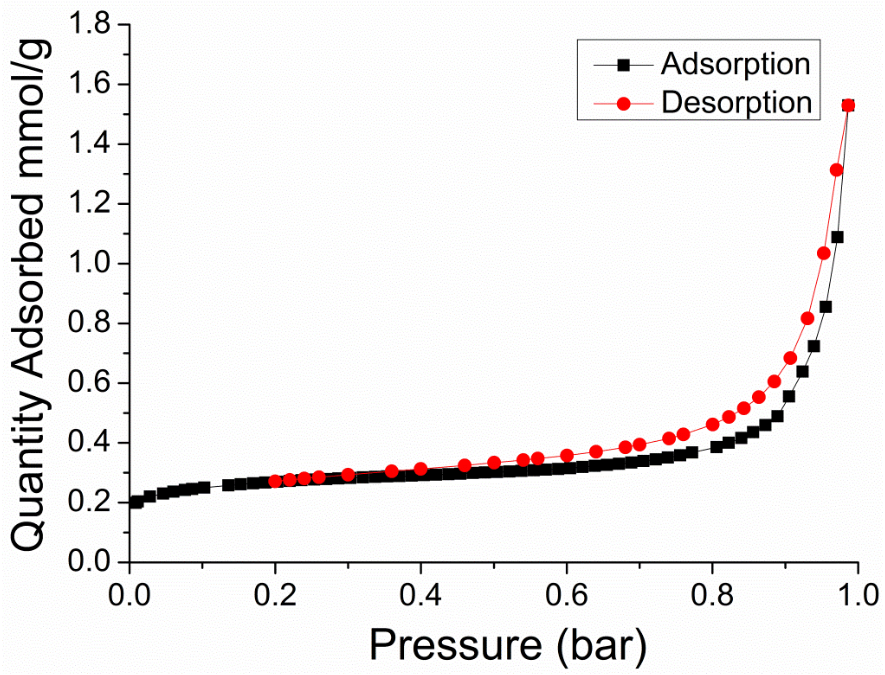

Figure S2. X-ray diffraction (XRD) patterns of nanometer-sized zeolite NaA samples synthesized with different amounts of methylcellulose (MC) added: (a) NZ040; (b) NZ060; (c) NZ080; (d) NZ100; (e) N020; (f) NZ010; (j) Z0.

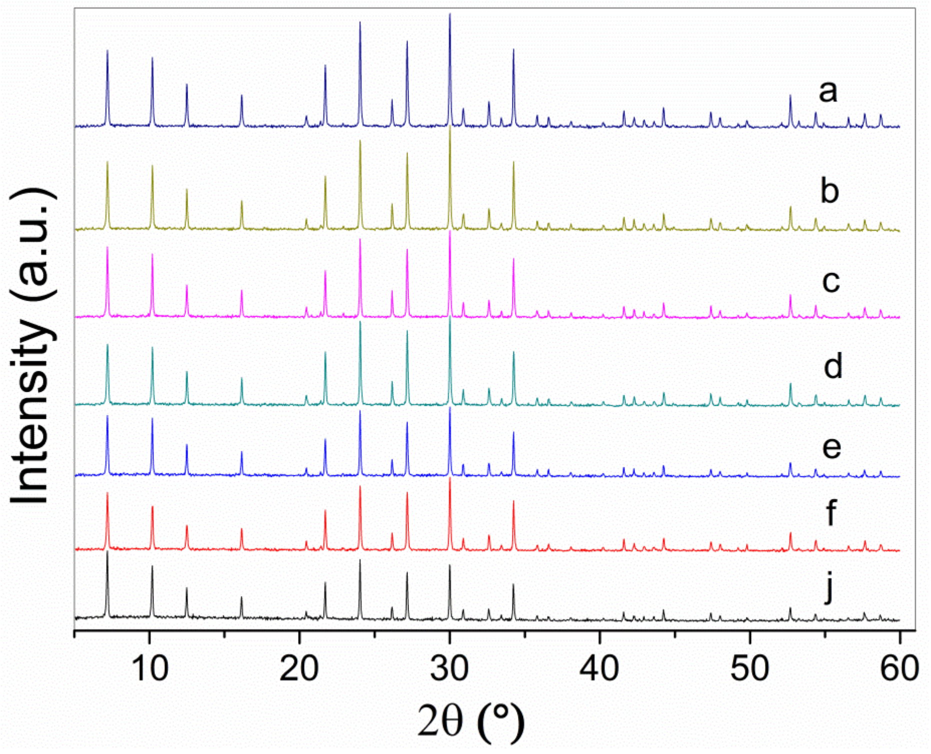

Figure S3. Comparison of the XRD patterns of synthesized zeolites A crystals with the addition of MC; (a) NZ040, and without any added MC; (b) Z0, compared with a commercial powder; (c) zeolite A.

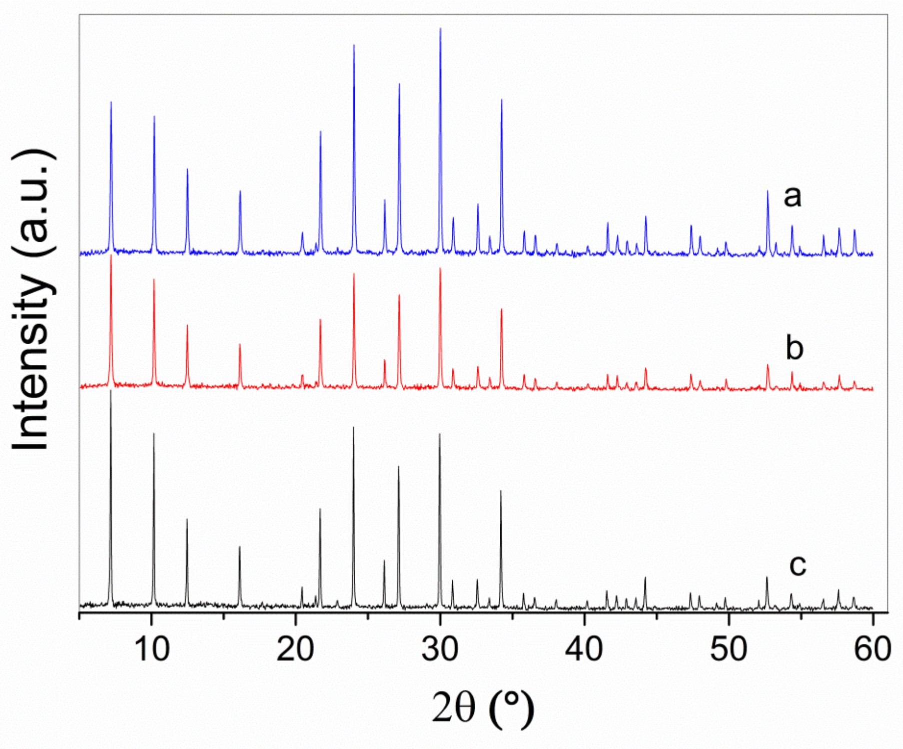

Figure S4. Elemental composition of nano- and micron-sized zeolites as obtained by energy dispersive X-ray spectroscopy (EDXS).

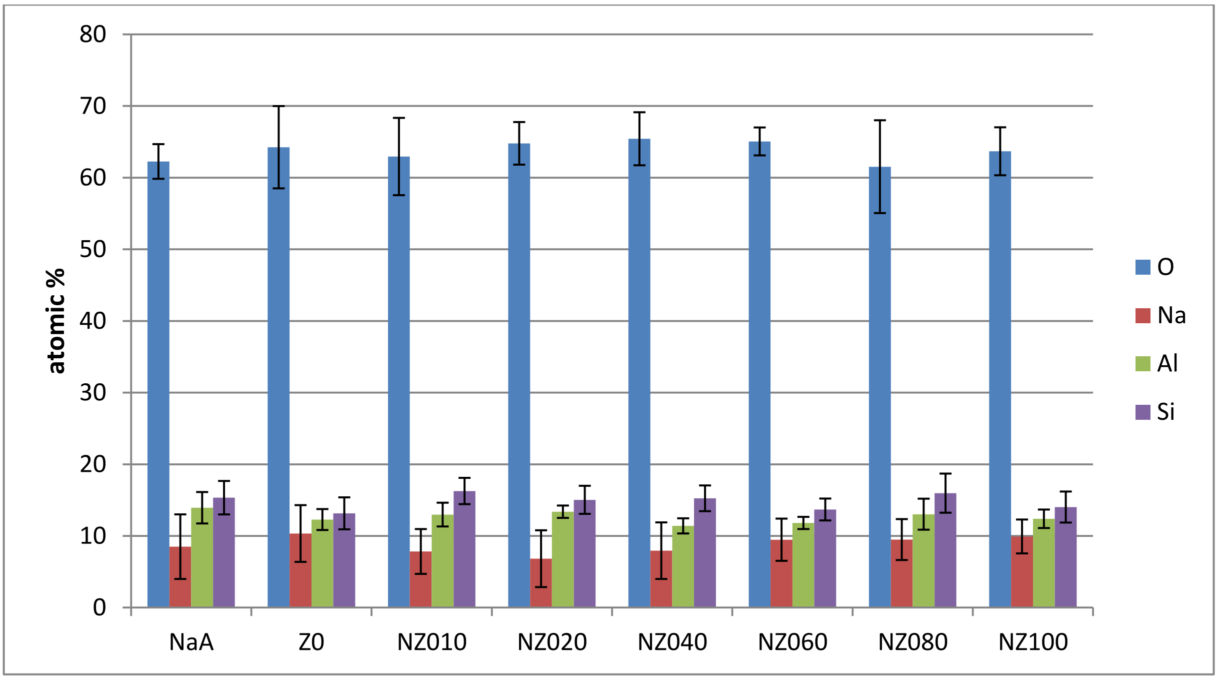

Figure S5. A logarithmic representation of remaining CO_2 adsorption capacity *versus* time of synthesized zeolite A nanocrystals.

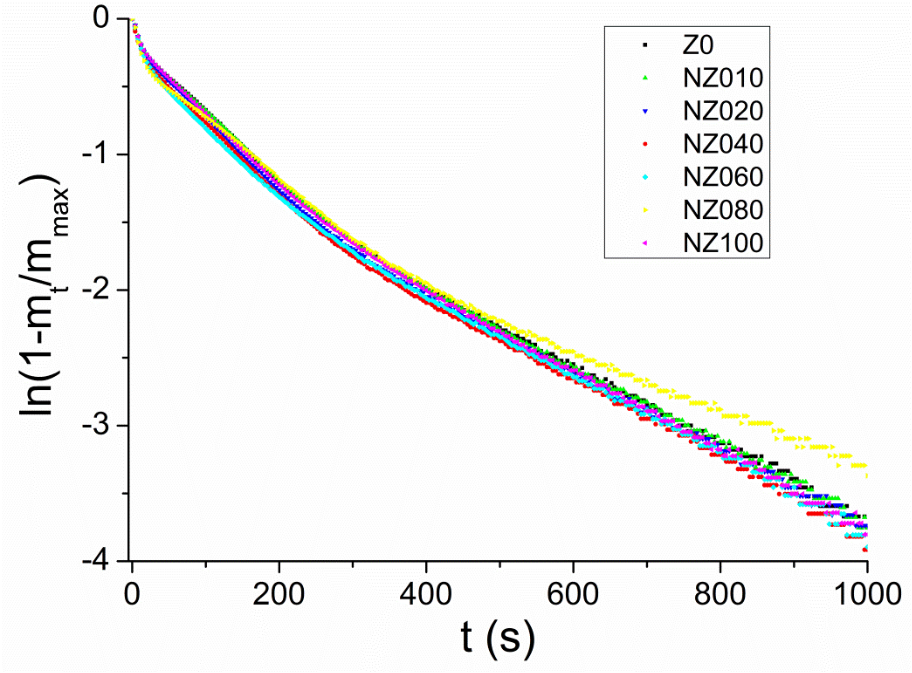

Figure S6. CO_2 adsorption capacity *versus* time of synthesized zeolite A nanocrystals.

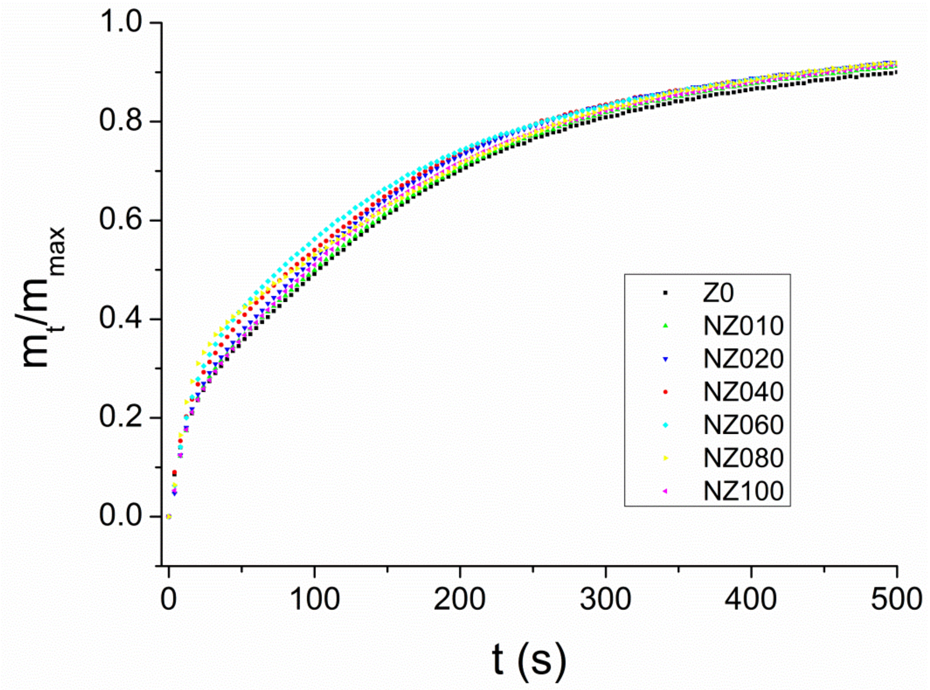

Supplement: Supplementary File 1 [file materials-07-05507-s001.pdf]
